# Identification of Possible Salivary Metabolic Biomarkers and Altered Metabolic Pathways in South American Patients Diagnosed with Oral Squamous Cell Carcinoma

**DOI:** 10.3390/metabo11100650

**Published:** 2021-09-23

**Authors:** Mariana de Sá Alves, Nayara de Sá Rodrigues, Celso Muller Bandeira, José Francisco Sales Chagas, Maria Beatriz Nogueira Pascoal, Gabrielle Luana Jimenez Teodoro Nepomuceno, Herculano da Silva Martinho, Mônica Ghislaine Oliveira Alves, Maria Anita Mendes, Meriellen Dias, Levy Anderson César Alves, Janete Dias Almeida

**Affiliations:** 1 Department of Biosciences and Diagnosis, Institute of Science and Technology, São Paulo State University (Unesp), São José dos Campos 12245-000, Brazil; mariana.sa@unesp.br (M.d.S.A.); nayarasarodrigues@hotmail.com (N.d.S.R.); bandeiracmu@yahoo.com.br (C.M.B.); 2 Department of Head and Neck Surgery, Hospital of the Pontifical Catholic University of Campinas, Campinas 13086-900, Brazil; josefcochagas@ig.com.br (J.F.S.C.); maribibia@ig.com.br (M.B.N.P.); 3 Centro de Ciências Naturais e Humanas (CCNH), Universidade Federal do ABC, Av. dos Estados, 5001, Santo André 09210-580, Brazil; gabrielle.lujimenez@gmail.com (G.L.J.T.N.); herculano.martinho@ufabc.edu.br (H.d.S.M.); 4 School of Dentistry, Universidade Mogi das Cruzes, Mogi das Cruzes 08780-911, Brazil; mgoliveiraalves@gmail.com; 5 School of Medicine, Anhembi Morumbi University, São José dos Campos 03164-000, Brazil; 6 Dempster MS Lab, Department of Chemical Engineering, Polytechnic School, University of Sao Paulo, Sao Paulo 05508-040, Brazil; mariaanita.mendes@gmail.com (M.A.M.); meriellend@gmail.com (M.D.); levyanderson@alumni.usp.br (L.A.C.A.)

**Keywords:** metabolomics, biomarkers, metabolites, oral squamous cell carcinoma, oral cancer, saliva, mass spectrometry, GC-MS

## Abstract

Oral squamous cell carcinoma (OSCC) represents 90% of oral malignant neoplasms. The search for specific biomarkers for OSCC is a very active field of research contributing to establishing early diagnostic methods and unraveling underlying pathogenic mechanisms. In this work we investigated the salivary metabolites and the metabolic pathways of OSCC aiming find possible biomarkers. Salivary metabolites samples from 27 OSCC patients and 41 control individuals were compared through a gas chromatography coupled to a mass spectrometer (GC-MS) technique. Our results allowed identification of pathways of the malate-aspartate shuttle, the beta-alanine metabolism, and the Warburg effect. The possible salivary biomarkers were identified using the area under receiver-operating curve (AUC) criterion. Twenty-four metabolites were identified with AUC > 0.8. Using the threshold of AUC = 0.9 we find malic acid, maltose, protocatechuic acid, lactose, 2-ketoadipic, and catechol metabolites expressed. We notice that this is the first report of salivary metabolome in South American oral cancer patients, to the best of our knowledge. Our findings regarding these metabolic changes are important in discovering salivary biomarkers of OSCC patients. However, additional work needs to be performed considering larger populations to validate our results.

## 1. Introduction

Oral cancer refers to the set of malignant neoplasms that affect the lips and other intraoral regions [1]. It represents the 16th most common neoplasm in the world, with 355,000 new diagnoses and 177,000 deaths in 2018 [2]. It is a highly relevant problem for global public health since there is no evidence of significant improvement for fast treatment and prevention in spite of all the progress in current research and therapies [3]. Among oral malignancies squamous cell carcinoma (OSCC) is the most prevalent histological type representing approximately 90% of cases. OSCC is often preceded by the presence of oral potentially malignant disorders. They are clinically identifiable as either white or red patches known as leukoplakia and erythroplakia, respectively. Non-healing ulcers may also be noticed along cancer development [4]. The highest incidence of OSCC occurs in the middle-aged population although the number of young individuals diagnosed with the disease has increased [5,6]. The most common site for OSCC is the tongue followed by the floor of the mouth. Less common sites include the gingiva, buccal mucosa, labial mucosa, and hard palate [4]. OSCC has a survival rate of approximately 80% for individuals detected with early stage disease (stage I) when compared to a rate of 20–30% in patients diagnosed at advanced stages (stages III–IV) [7]. This fact emphasizes the importance of early diagnosis. Unfortunately about 50% of cases are diagnosed in advanced stages (III and IV) [8,9] which implies a worse prognosis, increased costs, and a high mortality rate [10,11].

The predominant etiological factors for oral cancer are well established in the literature and include the use of tobacco and alcohol which act as carcinogenic substances responsible for constituting the so-called “field cancer” [12]. The carcinogenesis process is complex, being influenced by genetic and epigenetic alterations [13,14]. The fact is that the sooner these changes are detected, the earlier the disease will be discovered, contributing to a better prognosis for patients [15]. Conventional biopsy is considered the gold standard for the diagnosis of OSCC. However, it is inconvenient for large population screening and monitoring of patients due to its invasiveness, high cost, and need for trained personnel and equipment [16]. Thus, it is important to investigate biological molecules acting as biomarkers that may provide valuable diagnostic data on OSCC [14].

The search for biomarkers for chronic diseases and malignant neoplasms is a very active field of research worldwide [17]. Metabolomics employ state-of-the-art analytical techniques to recognize and study metabolic alterations in individuals who are undergoing some pathophysiological process or are undergoing pharmacological interventions and genetic modifications [18,19,20].

Biofluids—urine, blood, and saliva—are often used as clinical specimens of patients for metabolomic analysis [15]. Saliva is an oral fluid capable of reflecting the oral and systemic health conditions of individuals [21]. It is a complex and valuable composition that includes proteins, peptides, nucleic acids, enzymes, hormones, antibodies, electrolytes, antimicrobial constituents, growth factors, and other molecules associated with the phenotype and even diseases of individuals [22,23,24,25]. The main functions of saliva are related to digestion, swallowing, tasting, and lubrication of the oral mucosa. However, it is known that in addition to these functions saliva acts as a protective substance against pathogens and toxins due to its specific composition [26].

Previous studies have identified metabolomic biomarkers for OSCC [27,28,29,30,31,32,33,34,35,36,37,38,39,40,41,42,43]. Some of these probed salivary metabolites [27,28,29,30,31,32,33,34,35,36]. The fact that the metabolites profile can be influenced by the sample collection time [31,44], the food intake [28,30,31,45], the general oral health status [46], and even the oral microbiome [38,47] represents a challenge for standardization of salivary studies in order to avoid inconsistencies and reproducibility drawbacks. Ethnicity has also been shown to play an important role in the differentiation of metabolites since populations of distinct ethnicities presented distinct salivary metabolic profiles [31,37]. Most studies involving salivary metabolome in OSCC patients come from Asian individuals [38], showing the importance of studying different ethnic groups [48].

However, despite existing limitations, previous studies have shown consistent changes between OSCC and healthy patients [28], mainly due to the direct contact between saliva and the oral cancer lesion [30]. 

In the present work we investigated the salivary metabolites profile from a sample of OSCC patients from a South American population. The objectives were to identify possible salivary metabolomic biomarkers and also altered metabolic pathways.

## 2. Results

### 2.1. Demographic Data

The main clinical data of patients are summarized in Table 1. Data on sex and age did not show a statistically relevant difference between the groups (*p* < 0.05).

Table 2 presents the TNM cancer staging system, smoking habits, and racial ethnicity data of the patients.

### 2.2. Metabolomic Analysis

**Figure 1:**
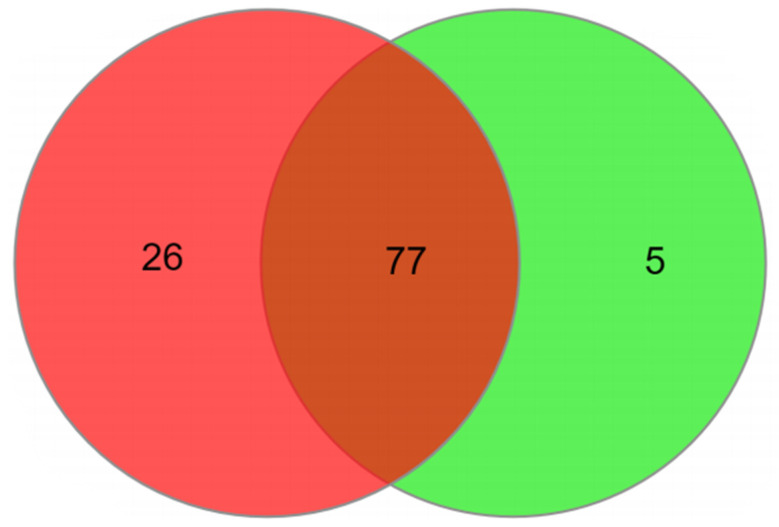
Venn diagram for salivary metabolites probed on OSCC (red) and control (green) groups.

A total of 108 metabolites were identified as relevant for OSCC and control discrimination. All metabolites found in both groups studied were allocated on a Venn diagram to assess their distribution between groups (Figure 1). The analysis showed that the OSCC group has a higher number of specific metabolites (26 metabolites), while the control group had 5 specific metabolites. Seventy-seven metabolites were common for both groups. These metabolites are show on Table 3.

**Figure 2:**
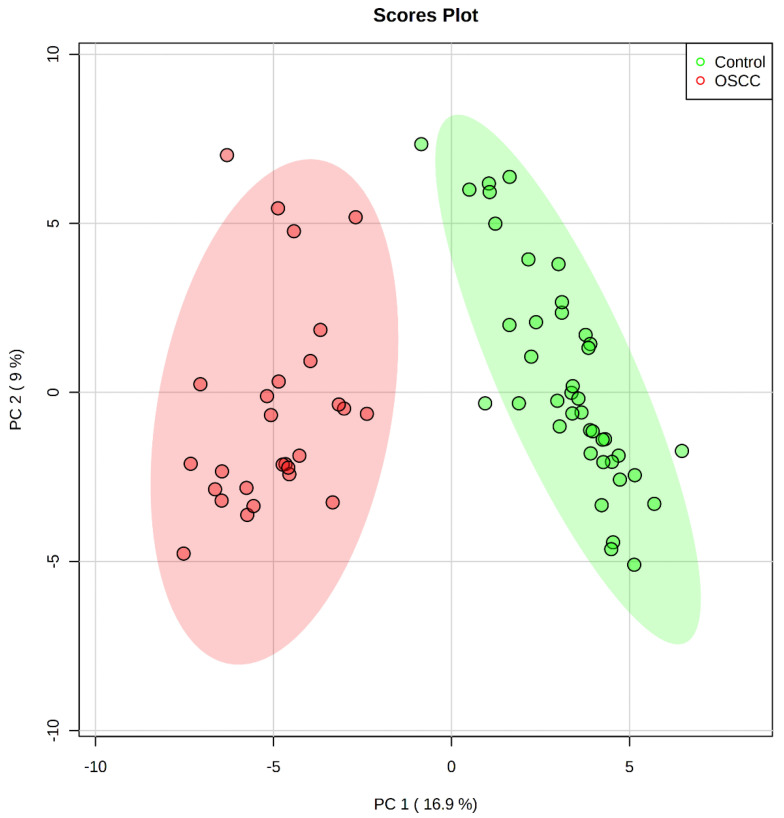
PCA score plot OSCC (green) and control (red) groups. Ellipses represent the loci of maximum variance of data.

The dispersion score plot PC2 against PC1 (Figure 2) shows a clear separation among groups.

**Figure 3:**
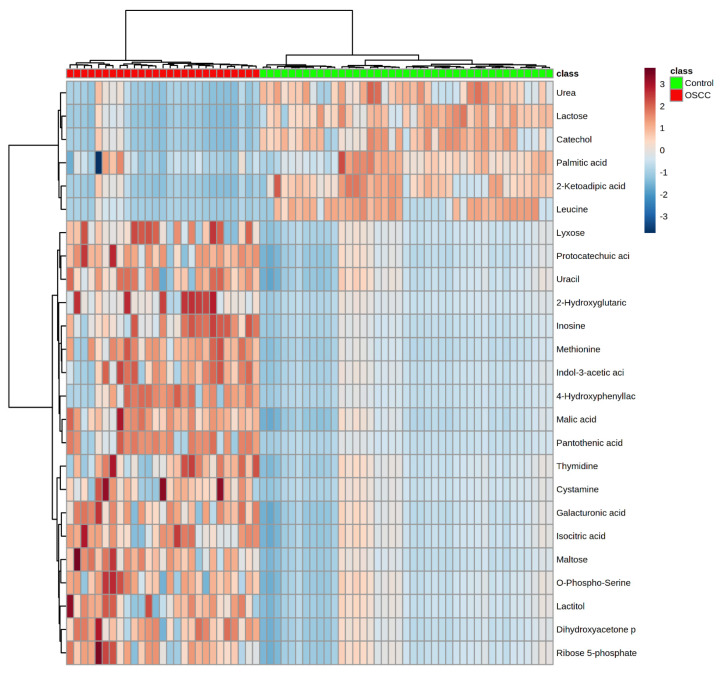
Heatmap using PCA data for OSCC and control classes.

The heatmap showing the clustering of classes against metabolites is shown in Figure 3. Samples in OSCC and control classes clustered in two big groups with 100% discrimination. Metabolites urea, lactose, catechol, palmitic acid, 2-ketoadipic acid and leucine appeared underexpressed in the OSCC group. On the other hand, lyxose, protocatechuic acid, uracil, 2-hydroxyglutaric, inosine, methionine, indol-3-acetic acid, 4-hydroxyphenyllac, malic acid, pantothenic acid, isocitric acid, maltose, O-phospho-serine, lactitol, dihydroxyacetone and ribose 5-phosphate were overexpressed in patients with cancer.

Table 4 displays the up- and down-regulated metabolites presenting statistical relevance. Twenty metabolites were up-regulated (malic acid, methionine, maltose, protocatechuic acid, inosine, pantothenic acid, dihydroxyacetone phosphate, hydroxyphenylatic acid, galacturonic acid, indole-3-acetic acid, uracil, isocitric acid, ribose-5-phosphate, o-phospho serine, lactitol, gluconic acid, hippuric acid, 3-hydroxypropionic acid and spermidine) and 20 down-regulated (lactose, catechol, 2-ketoadipic acid, leucine, urea, maleic acid, palmitic acid, ornithine, margaric acid, sucrose, octadecanol, threitol, acetoacetic acid, methionine sulfone, phosphoric acid, elaidic acid, mannose, sorbitol, citric acid, 3-aminopropanoic acid) in OSCC samples.

### 2.3. Analysis of Altered Metabolic Pathways in the OSCC Group

The precedent analysis enables us to investigate the altered metabolic pathways in OSCC patients and find the role of each metabolite in these pathways.

**Figure 4:**
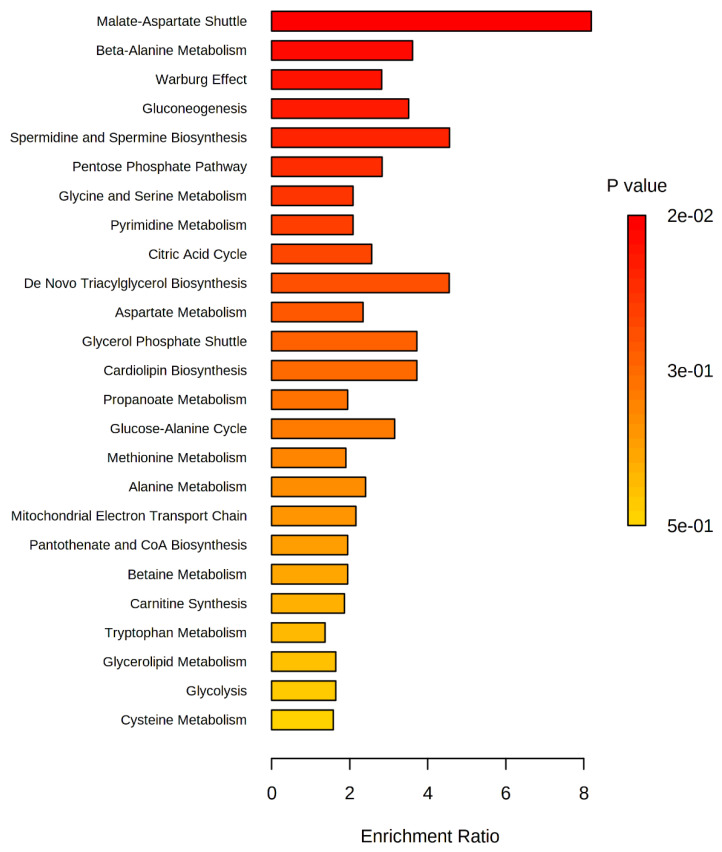
Statistically significant (*p* < 0.05) for OSCC.

We analyzed 25 metabolites which were found exclusively in the OSCC group. Cystamine was absent from the databases of the chosen metabolomic compound and was excluded from further analysis. Thus, the role of 2-ketoglutaric acid, 2-hydroxyglutaric acid, 3-hydroxypropionic acid, 4-hydroxyphenylatic acid, galacturonic acid, gluconic acid, hippuric acid, indol-3-acetic acid, isocitric acid, malic acid, pantothenic acid, protocatechuic acid, ureidosuccinic acid, spermidine, dihydroxyacetone phosphate, inosine, lactitol, lyxose, maltose, methionine, O-phospho-serine, ribose 5-phosphate, sorbose, thymidine, and uracil in metabolic pathways was investigated. The pathway enrichment analysis is shown in Figure 4.

A total of 41 metabolic pathways were identified as present in OSCC salivary samples. However, only 25 presented statistical relevance. From these we can mention the malate-aspart (*p =* 0.0229), beta-alanine metabolism (*p =* 0.0467), and the Warburg effect (*p =* 0.048) signaling pathways.

### 2.4. Analysis of Possible Salivary Biomarkers for the OSCC Group

A receiver operating characteristic (ROC) curve was used to establish promising biomarkers for OSCC. The area under the ROC curve value (AUC) measures the performance of the biomarkers. Thus, an excellent biomarker has an AUC value of 1.0. Good biomarkers have AUC > 0.80. Using this criterion, we list in Table 5 the set of possible good salivary biomarkers for OSCC.

## 3. Discussion

The relevance of the investigation of the salivary metabolome of OSCC relies on the identification of predominantly altered metabolic pathways which may lead to the discovery of possible biomarkers. This could improve the capacity of early diagnosis and, consequently, the quality of life of patients.

Reports of the salivary metabolome of patients with oral cancer described in the literature are presented in Table 6 and compared to our findings. The present study sought the main altered salivary metabolic pathways in OSCC patients and, additionally, the main metabolites that can be used as future salivary biomarkers for early diagnosis. To the best of our knowledge, this is the first research in this area focusing on Latin American patients.

OSCC is mostly diagnosed at late stages, as also evidenced by our study, in which only 15% of patients were diagnosed with early-stage cancer (stage I), revealing that early diagnosis remains a challenge [49]. It is noteworthy that early diagnosis implies greater possibilities of successful treatment, less mutilation of the patient concerning the treatments carried out, decreased mortality rate, and reduced costs [50,51,52].

### 3.1. The Malate-Aspartate Shuttle Pathway

ATP consumption is higher for cancer cells compared to healthy ones. Thus, high glycolytic rates and mitochondrial oxidative phosphorylation are observed in tumor cells to deliver a greater amount of ATP in a short period of time [53]. Glycolysis is the metabolic pathway chosen by the body in the absence of oxygen, so less energy is generated, although the pathway normally occurs without the presence of oxygen [54]. If the body has plenty of oxygen, the glycolysis process will only be the beginning of the aerobic respiration cycle, in which the lactate generated by glycolysis will be consumed by the tricarboxylic acid cycle, also known as the Krebs cycle [55]. During the Krebs cycle, ATP is not produced directly. NADH and FADH2 are produced, which are essential for the production of ATP during oxidative phosphorylation. Oxidative phosphorylation is the preferred method of generating energy in the presence of oxygen, since the process generates 38 ATPs in contrast to anaerobic glycolysis that generates 2 ATPs [56].

The tricarboxylic acid cycle (Krebs cycle) and oxidative phosphorylation occur within the mitochondria. The malate-aspartate shuttle is responsible for transporting NADH from the cytoplasm to the mitochondrial matrix in the ATP production process [57,58]. In our study, the malate-aspartate launcher is one of the altered pathways and it is directly related to the energy production of tumor cells. The malic acid metabolite was abundant in most patients with OSCC. Malic acid is an intermediate product of the Krebs cycle explaining its higher concentration in patients with OSCC [59]. Based on our results, there is a high energy production in cancer cells that provides a favorable environment for disorderly growth.

### 3.2. Warburg Effect Pathway

Cancer metabolism has been studied for decades, mainly because cells exhibit rapid growth, proliferation, and survival [60]. These characteristics are inherent in an altered metabolism. In 1920, Otto Warburg observed a common characteristic in the metabolism of tumor cells. This characteristic consisted of increased glucose uptake with high lactate release, signaling that the rate of glycolysis in tumor cells is high even in the presence of oxygen and perfectly functioning mitochondria [61]. This process, known as the Warburg effect, was established as the form of energy generation in tumor cells [60,61,62,63]. Our findings indicate that the Warburg effect was also one of the metabolic pathways activated in OSCC patients.

Although the Warburg effect is established as the way tumor cells acquire energy, some studies have shown that several types of cancer can obtain energy through oxidative phosphorylation in conjunction with glycolysis. A study on breast cancer metabolism reported that 20% of energy production comes from the glycolytic pathway and 80% from oxidative phosphorylation [64]. Furthermore, in a study on hepatoma cells, it was found that the cells obtained energy mainly through the oxidative pathway, in contrast to a small portion via the glycolytic pathway [65].

Xu and Guppy conducted a study with different types of cancers (breast, ovary, lung, uterus, melanoma, various types of hepatomas, and many others) to measure the rate of ATP production through glycolytic and oxidative processes. Among the types of cancer studied, the average contribution of the glycolytic pathway to the production of ATP was 17%. The authors concluded that the vast majority of tumor cells can generate ATP via oxidative phosphorylation, but also through glycolysis, in addition to the fact that some tumors are glycolytic as a result of the hypoxic environment [66]. This corroborates our findings since both the Warburg effect and malate-aspartate pathways contributed to the maintenance of oxidative phosphorylation. Studies involving OSCC show that the Warburg effect is present in cell metabolism [28,30,34,36,37]. However, our study is the pioneer in demonstrating that oxidative phosphorylation is also present in OSCC.

### 3.3. Beta-Alanine Pathway

Beta-alanine is a non-essential amino acid responsible for reducing fatigue and increasing muscle strength [67,68]. Its metabolism was indirectly involved with uracil and spermidine metabolites [69], both up-regulated in OSCC. A previous study on oral cancer metabolome revealed the beta-alanine metabolite as a possible biomarker for oral cancer [31]. It is related to conditions of hypoxia, hypoglycemia, ischemia, and oxidative stress due to the presence of free radicals [70]. Therefore, this oxidative stress is responsible for damage to neural cells [71]. In this sense, a study on metabolites in breast cancer demonstrated that beta-alanine is one of the metabolites related to high glycolytic activity and associated with aggressiveness of tumor cells [72].

### 3.4. Biomarkers

We have found that some metabolites such as malic acid, maltose, methionine, and inosine were over-expressed in the saliva of patients with OSCC. Malic acid was reported above to be present in the malate-aspartate pathway. It has been reported that maltose is a possible natural substance with carcinogenic potential [73].

Ishikawa et al. identified the following metabolites over-expressed in the saliva of OSCC patients: hypoxanthine, guanine, guanosine, trimethylamine N-oxide, spermidine, pipecolate, methionine [28]. Of these, methionine and spermidine were also increased in our study, with an AUC of 0.92 and 0.80, respectively. Ishikawa et al. showed that the metabolism of purines was altered since the metabolites hypoxanthine, guanine, and guanosine are part of this pathway. However, in our study, only the inosine metabolite was altered in the purine metabolism pathway, being a potential biomarker for OSCC.

Ohshima et al., also found urea in lower concentrations in OSCC patients and in higher concentrations in control patients, indicating that the urea cycle might be altered in oral cancer. This was the first study to describe urea as a possible biomarker for oral cancer. It was carried out using capillary electrophoresis-mass spectrometry (CE-MS) to evaluate saliva from Japanese patients with OSCC [37]. Liang et al. observed changes in urea concentration when studying the metabolome of patients with gastric cancer [74]. Our study showed a significant change in urea in salivary samples from control patients, corroborating data from the Japanese survey [37]. According to Ohshima et al., OSCC patients may have difficulty eating due to pain and trouble in opening the mouth, which makes it difficult to ingest proteins and, consequently, form urea [37]. In addition, these patients may have a *Helicobacter pylori* infection, which produces urease, reducing the availability of urea [75].

Another metabolite present in salivary metabolome studies found in our study is leucine. Leucine was present in a study with 24 salivary metabolites that are candidate biomarkers in OSCC [31]. Wei et al. identified leucine, isoleucine, valine, and all intermediate branched-chain amino acids (BCAAs) underexpressed in the saliva of OSCC patients [36]. These metabolites are involved in the Krebs cycle. Thus, the activation of the glycolytic pathway (Warburg effect) decreases the entry of pyruvate into the TCA cycle. Therefore, the aforementioned metabolites are less necessary for the process due to the lack of energy supply via TCA [36].

In summary, we conclude that the whole nature of cellular energy production was altered in the OSCC group. This is the first salivary metabolomic study of a South American population with OSCC. Therefore, carrying out new studies covering larger populations may bring similar results and new insights so that these metabolites can be used as a non-invasive tool in oral cancer screening. Thus, salivary metabolic screening in populations exposed to risk factors, such as smoking and alcohol consumption, can reveal possible salivary biomarkers of oral cancer and improve the early diagnosis of carcinoma.

## 4. Materials and Methods

This study was conducted in accordance with the Declaration of Helsinki, and the protocol was approved by the Research Ethics Committee of the Institute of Science and Technology of São José dos Campos (ICT-UNESP), as part of the study entitled “Genetic study of the main risk factors in the prognosis of patients with oral squamous cell carcinoma”, protocol number 1.033.312/2015 PH/CEP. Patients were informed about the objectives, propositions, and conditions of this project, and those who agreed to participate signed the Free and Informed Consent Term (FICT). After acceptance, all patients underwent an extra and intraoral physical examination. Patients were divided into OSCC and control groups.

The OSCC group consisted of 27 patients diagnosed with OSCC. Inclusion criteria were patients over 18 years of age concomitant with the diagnosis of OSCC. The exclusion criterion considered patients diagnosed with cancer anywhere on the body that had already undergone some type of treatment, that is, surgery, radiotherapy, and chemotherapy. Cancer staging followed tumor-node-metastasis (TNM) classification according to the 8th edition of the American Joint Committee on Cancer (AJCC) Cancer Staging Manual [76]. The control group was composed of 41 patients from the oral medicine outpatient clinic of the Department of Biosciences and Oral Diagnosis of ICT-UNESP. The inclusion criterion was patients over 18 years of age, who wanted to participate in the research. The exclusion criterion was patients with some type of cancer during their lifetime.

### 4.1. Collection and Storage of Salivary Samples

Patients were instructed not to ingest pasty or hardened foods for 1 h before collection, as well as not to consume alcoholic beverages for at least 12 h before saliva collection. They could only swallow water and had to brush their teeth at least 2 h before the collection. Patients were instructed to expectorate 3 mL of saliva in the plastic tubes, which were then hermetically closed, immersed in ice, and transported within 1 h to the storage location. Salivary samples were stored in a freezer at −80 °C at the Laboratory of Microbiology and Immunology of the ICT-UNESP.

### 4.2. Preparation and Metabolomic Analysis of Salivary Samples

The methodology for analyzing the salivary metabolome was adapted from a previous study [77]. First, 300 µL of saliva samples were dried in a vacuum centrifuge (Labconco Centrivap Concentrator, Kansas City, MI, USA). Metabolites were extracted adding 300 µL of methanol containing methionine sulphonate as internal standard and stirred (LCG Vortex Mixer, Taiwan, China) for 2 min, and the supernatant was dried in a vacuum centrifuge. After extraction, derivatization was performed by adding 100 µL of a solution with proportions (1:1) of N-methyl-N-(trimethylsilyl) trifluoroacetamide and a solvent solution: acetonitrile/dichloromethane/cyclohexane (5:4:1) and 5% trimethylamine. The samples were stirred for 30 s and then kept in a thermal bath (Nova Instruments NI 1225, Piracicaba, Brazil) at 60 °C for 1 h. Next, the samples were centrifuged (Eppendorf MiniSpin, Hamburg, Germany) at 12,044× *g* for 2 min. The supernatant was analyzed via GC-MS. The data obtained were processed using GCMS solution and the metabolites identified using Smart metabolite database version 4.2. GC-MS analysis conditions:

- MRM analysis method
- running time: 67 min
- injection temperature: 280 °C
- interface temperature: 280 °C
- ionization source temperature: 200 °C
- heating rate: from 100 °C to 320 °C in a linear ramp of 4 °C/min, remaining at this temperature for 8 min.

### 4.3. Statistical Analysis

Clinical data were analyzed from the description of categorical variables with counts and proportions and quantitative variables with normal and asymmetric distribution and described as mean and deviation. For the consideration of normality, visual inspection of histograms or application of a normality test was used when appropriate. For all analyses, we considered the significance level of 5% (*p* < 0.05). Descriptive statistical analysis of clinical data was performed using GraphPad Prism 5.03 software (GraphPad Software, San Diego, CA, USA). For the salivary metabolomic analysis, principal component analysis (PCA) was performed, which allows the amount of information collected to be reduced. Its results select a subset of n variables capable of describing variability of data. The heatmap cluster was also used as a way to visualize the metabolites and hierarchical grouping of the compounds in each group. To demonstrate the significance of the metabolic data, we used the Wilcoxon-Mann-Whitney test. The *p* values to assess differences in metabolite concentrations between oral cancer and controls were corrected using the false discover rate (FDR) analysis of Benjamin-Hockberg [78] to consider several independent tests at a value of *q* < 0.05.

The volcano plot was used to visibly identify and illustrate the metabolites that are significant and most expressed in each study group. The volcano plot presents a diagram showing the set of metabolites in the salivary samples that would be down- and up-regulated. That is, it can indicate whether the compound is present with significance in the control group or in the OSCC group. The volcano plot combines the measure of statistical significance, in this case the q-value (FDR) with the measure of magnitude variation FC (fold change). In order to identify possible salivary biomarkers for OSCC, a ROC (receiver operating characteristic) curve was drawn for each metabolite. For this, the ROC curve uses the parameters of sensitivity and specificity. The area under the ROC curve, also called AUC, allows identification of whether a condition is present or not. That is, an AUC of 0.5 has no discriminating capacity, while an AUC of 1.0 shows an ideal discrimination [79]. For our study, we considered AUCs above 0.8 as the ideal cutoff point. In addition to the statistical tests mentioned above, the averages and standard deviations of the metabolites of each group were also performed. MetaboAnalyst 5.0 software (https://www.metaboanalyst.ca/ accessed on 18 December 2020) was used to analyze the metabolomic data.

All metabolites found were allocated on a Venn diagram to assess their distribution between groups. InteractiVenn (http://www.interactivenn.net/ accessed on 18 December 2020) was used for the analysis of the Venn diagram. The relative quantification of the metabolites for each group was performed from specific peak areas for each metabolite using the MRM analysis method. For the search for metabolites to be effective in the main databases, the Kyoto Encyclopedia of Genes and Genomes (KEGG pathway) and Small Molecule Pathway Database (SMPDB), the initial standardization of compound names was carried out on the MetaboAnalyst platform.

## 5. Conclusions

In summary, in our study, three important altered metabolic pathways were identified in OSCC for South American patients: the malate-aspartate shuttle, the beta-alanine metabolism pathway and the Warburg effect. These pathways are related to the cellular energy production in carcinogenesis, promoting a favorable environment for high energy consumption and cell survival. It was possible to statistically distinguish the salivary metabolites of control patients compared to patients with oral cancer. These metabolic changes may help in the discovery of salivary biomarkers of oral cancer and stimulate interest for new studies with larger populations to validate our results.

## Figures and Tables

**Table 1:** Demographic data of patients.

Variable	OSCC ^1^ (*n* = 27)	CONTROL (*n* = 41)	*p*-Value *
Sex ^2^			
Female	8 (29.6%)	20 (49%)	0.3326
Male	19 (70.4%)	21 (51%)	0.9131
Age ^3^	57 ± 13.87	57.34 ± 11.66	0.9131
	(28–88)	(31–86)	

^1^ OSCC, oral squamous cell carcinoma group. ^2^ Sex was described with their respective means and (%) percentages. ^3^ Age described as mean ± standard deviation and in parentheses the minimum and maximum age of the patients. n represents the number of patients in each group. * *p*-values according to the Student’s *t*-test considering as significant *p* < 0.05.

**Table 2:** Cancer staging system, smoking habits, and racial ethnicity of patients.

TNM ^1^	OSCC (*n* = 27)	Control (*n* = 41)
T (tumor)		
T1	5 (19%)	
T2	7 (26%)	Not applicable
T3	6 (22%)	
T4	9 (33%)	
N (node)		
N0	14 (52%)	
N1	4 (15%)	Not applicable
N2	8 (30%)	
N3	1 (4%)	
M (metastasis)		
M0	27 (100%)	Not applicable
Stages		
I	4 (15%)	
II	4 (15%)	Not applicable
III	6 (22%)	
IV	13 (48%)	
Smokers	20 (74%)	8 (20%)
Non smokers	7 (26%)	20 (49%)
Ex smokers	0 (0%)	13 (32%)
Racial ethnicity		
Leucoderma	24 (89%)	32 (78%)
Melanoderm	1 (4%)	4 (10%)
Pheoderm	2 (7%)	4 (10%)
Xanthoderm	0 (0%)	1 (2%)

^1^ TNM—classification of malignant tumors. The TNM system is used to describe the anatomical extension of the disease, where T—the extension of the primary tumor, N—the absence or presence and extension of metastasis in regional lymph nodes, M—the absence or presence of distant metastasis. All data are described with their respective n of each group and their respective (%) percentages.

**Table 3:** Exclusive and shared salivary metabolites for OSCC and control groups.

OSCC	CONTROL	OSCC AND CONTROL
2-Hydroxyglutaric acid	2-Ketoadipic acid	1,6-Anhydroglucose
2-Ketoglutaric acid	Catechol	1-Hexadecanol
3-Hydroxypropionic acid	Lactose	2-Aminoethanol
4-Hydroxyphenyllactic acid	Leucine	2-Deoxy-glucose
Cystamine	Urea	2-Hydroxyisovaleric acid
Dihydroxyacetone phosphate		3-Aminoglutaric acid
Galacturonic acid		3-Aminoisobutyric acid
Gluconic acid		3-Aminopropanoic acid
Hippuric acid		3-Hydroxyisovaleric acid
Indol-3-acetic acid		3-Phenyllactic acid
Inosine		4-Aminobutyric acid
Isocitric acid		5-Aminovaleric acid
Lactitol		Acetoacetic acid
Lyxose		Adenine
Malic acid		Allose
Maltose		Arabitol
Methionine		Arachidonic acid
O-Phospho-Serine		Arginine
Pantothenic acid		Aspartic acid
Protocatechuic acid		Batyl alcohol
Ribose 5-phosphate		Cadaverine
Sorbose		Caproic acid
Spermidine		Citramalic acid
Thymidine		Citric acid
Uracil		Cysteine
Ureidosuccinic acid		Dopamine
		Eicosapentaenoic acid
		Elaidic acid
		Fructose
		Galactosamine
		Galactose
		Glucono-1,5-lactone
		Glucosamine
		Glucose
		Glucuronic acid
		Glutamic acid
		Glycerol
		Glycerol 2-phosphate

**Table 4:** Set of metabolites up- and down-regulated in OSCC samples according to PCA analyses.

Metabolites	OSCC		Control		*p*-Value ^1^	*q*-Value (FDR) ^2^	FC	Volcano Plot ^3^
	Mean	Standard Deviation	Mean	Standard Deviation				
Lactose *	−1.090	0.492	0.718	0.673	<0.0001	3.1755 × 10^−16^	0.015832	Down
Malic acid **	0.917	0.622	−0.604	0.444	<0.0001	3.7012 × 10^−16^	40.712	Up
Methionine **	1.088	0.939	−0.717	0.367	<0.0001	3.0633 × 10^−15^	311.66	Up
Catechol *	−0.952	0.521	0.627	0.734	<0.0001	7.1635 × 10^−13^	0.035587	Down
2-Keto adipic acid *	−0.925	0.522	0.609	0.768	<0.0001	6.363 × 10^−12^	0.029706	Down
Maltose **	0.889	0.959	−0.586	0.407	<0.0001	2.0868 × 10^−11^	325.18	Up
Protocatechuic acid **	0.806	0.827	−0.531	0.447	<0.0001	2.7666 × 10^−11^	35.723	Up
Leucine *	−1.177	0.394	0.775	1.173	<0.0001	8.7168 × 10^−11^	8.2595 × 10^−4^	Down
Inosine **	1.070	1.317	−0.704	0.330	<0.0001	9.7882 × 10^−11^	2873.0	Up
Pantothenic acid **	1.153	1.459	−0.759	0.304	<0.0001	1.4172 × 10^−10^	4271.4	Up
Urea *	−0.861	0.530	0.567	0.810	<0.0001	1.687 × 10^−10^	0.037894	Down
Dihydroxyacetone phosphate **	0.793	0.895	−0.522	0.439	<0.0001	1.687 × 10^−10^	45.791	Up
4-hydroxyphenylactic acid **	1.092	1.403	−0.719	0.318	<0.0001	2.1476 × 10^−10^	2173.8	Up
Galacturonic acid **	0.725	0.831	−0.477	0.467	<0.0001	8.9307 × 10^−10^	19.383	Up
Indole-3-acetic acid **	0.906	1.242	−0.597	0.365	<0.0001	3.0805 × 10^−9^	341.04	Up
Uracil **	0.644	0.817	−0.424	0.491	<0.0001	3.04 × 10^−8^	10.819	Up
Isocitric acid **	0.665	0.885	−0.438	0.472	<0.0001	3.6657 × 10^−8^	20.802	Up
Ribose-5-phosphate **	0.647	0.969	−0.469	0.461	<0.0001	3.1666 × 10^−7^	41.912	Up
O-Phospho-Serina **	0.609	0.945	−0.401	0.474	<0.0001	9.548 × 10^−7^	17.64	Up
Lactitol **	0.630	1.061	−0.415	0.446	<0.0001	2.1547 × 10^−6^	41.538	Up
Gluconic acid **	0.609	1.101	−0.401	0.443	<0.0001	7.7433 × 10^−6^	183.99	Up
2-Ketoglutaric acid **	0.515	0.836	−0.339	0.512	<0.0001	1.3092 × 10^−5^	6.7421	Up
Hipuric acid **	0.518	0.888	−0.341	0.506	<0.0001	1.4925 × 10^−5^	7.3906	Up
Maleic acid	−0.664	1.049	0.437	0.817	<0.0001	3.294 × 10^−5^	0. 8093	Down
Palmitic acid	−0.430	0.657	0.283	0.551	<0.0001	3.3213 × 10^−5^	0.38165	Down
3-hydroxypropionic acid **	0.608	1.265	−0.400	0.411	0.0002	4.4319 × 10^−5^	202.32	Up
Spermidine **	0.481	0.887	−0.317	0.514	0.0001	5.3374 × 10^−5^	10.562	Up
Ornithine	−0.614	1.197	0.405	0.986	0.0003	0.0010593	0.33872	Down
Margaric acid	−0.453	1.055	0.298	0.648	<0.0001	0.0018846	0.28057	Down
Sucrose	−0.487	1.005	0.321	0.928	0.0002	0.0039383	0.25406	Down
Octadecanol	−0.310	0.666	0.204	0.628	0.0010	0.0064518	0.56165	Down
Threitol	−0.465	1.148	0.307	0.847	0.0012	0.0069549	0.37775	Down
Acetoacetic acid	−0.373	0.732	0.246	0.826	0.0024	0.0074047	0.25319	Down
Methionine sulfone	−0.306	0.767	0.202	0.582	0.0001	0.0085698	1.123	Down
Phosphoric acid	−0.374	0.806	0.246	0.968	0.0103	0.022159	0.12317	Down
Elaidic acid	−0.254	0.578	0.167	0.722	0.0134	0.038044	0.4826	Down
Mannose	−0.398	1.309	0.262	0.881	0.0324	0.042273	0.51969	Down
Sorbitol	−0.361	0.890	0.238	1.048	0.0173	0.046325	0.11612	Down
Citric acid	−0.416	1.200	0.274	1.111	0.0369	0.046725	0.11946	Down
3-Aminopropanoic acid	−0.324	0.895	0.213	0.907	0.0004	0.048905	0.39703	Down

^1^ *p*-value was calculated using the Wilcoxon-Mann-Whitney test (*p*-value < 0.05). ^2^ All metabolites shown in the table were statistically significant with a false discovery rate (FDR) of 5%. ^3^ Volcano plot shows up- and down-regulated metabolites in patients with OSCC. * Metabolites exclusively found in control patients. ** Metabolites exclusively found in OSCC patients.

**Table 5:** Area Under the Receiving—Operator Curve (AUC) for possible OSCC salivary biomarkers.

Metabolite	AUC
Malic acid	0.98103
Lactose	0.96387
Catecol	0.94670
2-ketoadipic acid	0.94128
Maltose	0.93360
Methionine	0.92502
Urea	0.92502
Leucine	0.92322
Inosine	0.92186
Protocatechuic acid	0.91192
Dihydroxyacetone phosphate	0.89657
Galacturonic acid	0.88573
Margaric acid	0.86902
Uracil	0.86721
Isocitric acid	0.86585
Ribose 5-phosphate	0.84146
O-Phospho-Serine	0.82385
Indole-3-acetic acid	0.82204
Palmitic acid	0.82204
2-ketoglutaric acid	0.81798
Maleic acid	0.81030
Pantothenic acid	0.80307
Spermidine	0.80217

**Table 6:** Main salivary metabolomic studies of patients with OSCC.

Possible Salivary Metabolic Biomarkers	Studied Population	Notes	References
Malic acid ↑, Lactose ↓, Catecol ↓, 2-Keto adipic acid ↓, Maltose ↑, Methionine ↑, Urea ↓, Leucine ↓, Inosine ↑, Protocatechuic acid ↑ and others metabolites present in Table 3	South American	We compared OSCC patients with healthy control	This study
Lactic acid ↑, phenylalanine ↓, valine ↓	Not mentioned in the study	They compared OSCC patients with healthy control and oral leukoplasia	[36]
L-phenylalanine ↓, L-leucine ↓, Propionylcholine ↑, Acetylphenylalanine ↓, sphinganine ↓, phytosphingosine ↓, S-carboxymethyl-L-cysteine ↓, Choline ↑, betaine ↑, pipecolinic acid ↑, L-carnitine ↓	Chinese	They compared OSCC patients with healthy control	[32,33,34,35]
S-adenosylmethionine ↑, pipecolate ↑	Not mentioned in the study	Two cases from the oral cancer group were oral melanoma	[28]
Ornithine ↓, o-hydroxybenzoate ↓, ribose-5-phosphate ↓	Caucasian, African American, Hispanic, Asian	They compared OSCC patients and oral epithelial dysplasia patients with the healthy control	[29]
Alanine ↑, choline ↑, Leucine + isoleucine ↑, glutamic acid ↑, 120.0801 m/z ↑, phenylalanine ↑, alpha-aminobutyric acid ↑, serine ↑	Caucasian, Asian, African-American, Hispanic	They compared OSCC patients with healthy control	[31]
Indole-3-acetate ↑, ethanolamine phosphate ↑	Not mentioned in the study	They compared OSCC patients with control patients with oral lichen planus	[27]
They studied conductive polymer spray ionization mass spectrometry (CPSI-MS) associated with machine learning (ML) as a viable tool for the diagnosis of OSCC	Chinese	They compared OSCC patients with oral lichen planus and oral leukoplakia controls	[30]

↑ Up arrow indicates increased metabolites in OSCC patients. ↓ Down arrow indicates decreased metabolites in OSCC patients.

## Data Availability

The data presented in this study are available on request from the corresponding author. The data are not publicly available due to the privacy of the patients assisted in the research.
